# A Novel Signature for Predicting Prognosis of Smoking-Related Squamous Cell Carcinoma

**DOI:** 10.3389/fgene.2021.666371

**Published:** 2021-04-22

**Authors:** Chang Chen, Xiaoqing Cheng, Shuyan Li, Huanghui Chen, Mengjing Cui, Linlin Bian, Hui Jin

**Affiliations:** ^1^Department of Epidemiology and Health Statistics, School of Public Health, Southeast University, Nanjing, China; ^2^Jiangsu Provincial Center for Disease Control and Prevention (Jiangsu Institution of Public Health), Nanjing, China; ^3^Anhui Province Veterans Hospital, Bengbu, China

**Keywords:** smoking cessation, homologous recombination deficiency, prognosis, SCC, risk score model

## Abstract

Tobacco smoking is an established risk factor for squamous cell carcinoma (SCC). We obtained smoking-related SCC, including cervical SCC (CSCC), esophageal SCC (ESCC), head and neck SCC (HNSC), and lung SCC (LUSC), from The Cancer Genome Atlas (TCGA) database to investigate the association between smoking status (reformed and current smoking) and prognosis. We found that reformed smokers had a better prognosis than current smokers in CSCC (*p* = 0.003), HNSC (*p* = 0.019), and LUSC (*p* < 0.01) cohorts. Then, we selected LUSC cohorts as the training cohort and other SCC cohorts as the test cohorts. Function analysis revealed that homologous recombination (HR) was the most significant pathway involved in smoking-induced LUSC. Moreover, the effect of cross-talk between the smoking status and HR deficiency (HRD) on the prognosis was further evaluated, revealing that quitting smoking with high HRD scores could significantly improve patients’ prognosis (*p* < 0.01). To improve prognosis prediction and more effectively screen suitable populations for platinum drugs and poly-ADP-ribose polymerase (PARP) inhibitors, we constructed a risk score model using smoking- and HRD-related genes in LUSC. The risk score model had high power for predicting 2-, 3-, and 5-year survival (*p* < 0.01, AUC = 0.67, 0.66, and 0.66). In addition, the risk scores were an independent risk factor for LUSC (HR = 2.34, 95%CI = 1.70–3.23). The practical nomogram was also built using the risk score, smoking status, and other clinical information with a good c-index (0.72, 95%CI = 0.70–0.74). Finally, we used other TCGA SCC cohorts to confirm the reliability and validity of the risk score model (*p* < 0.01 and AUC > 0.6 at 2, 3, and 5 years in CSCC and HNSC cohorts). In conclusion, the present study suggested that smoking cessation should be a part of smoking-related SCC treatment, and also provided a risk score model to predict prognosis and improve the effectiveness of screening the platinum/PARP population.

## Introduction

Squamous cell carcinomas (SCCs) originate from the epithelial tissues of the aerodigestive or genitourinary tracts. They often occur in the head and neck SCC (HNSC), esophageal SCC (ESCC), lung SCC (LUSC), and cervical SCC (CSCC), sharing common histological features and some risk factors. Among risk factors, tobacco smoking is an established risk factor for SCC, which can alter biological carcinogenesis pathways to promote cancer progression (Schuller, 2019; Sabbula and Anjum, 2020). Cigarette smoke contains many carcinogens that can cause genomic alteration and break immunologic homeostasis (Li et al., 2018). Many studies have reported that smoking cessation can eliminate the physiological driving force of cancer development and improve the prognosis of SCC patients (Dobson Amato et al., 2015; Yavorski and Blanck, 2016; Yang et al., 2020). However, the underlying mechanism of smoking-induced SCC remains unclear in smoking-related SCC.

Homologous recombination repair (HRR) is an important repair method for DNA double strand damage (Mackenroth and Alani, 2020). Cancer cells with homologous recombination deficiency (HRD) are sensitive to platinum drugs and poly-ADP-ribose polymerase (PARP) inhibitors. At present, BRCA1/2 mutation is the most comprehensive HRD biomarker (Hoppe et al., 2018). However, Turner and Ashworth proposed the concept of “BRCAness” to describe HRD without BRCA mutation but with a phenotype similar to BRCA mutation (Turner et al., 2004; Lord and Ashworth, 2016). Therefore, better biomarkers should be identified to screen a more effective platinum/PARP population. HRD scores, which are comprehensively calculated based on loss of heterozygosis (LOH), telomeric-allelic imbalance (TAI), and large-scale state transitions (LST), are considered as biomarkers of genomic instability with mutation (Takaya et al., 2020), which were applied in drug efficacy and tumor susceptibility evaluation (do Canto et al., 2019; Min et al., 2020).

In the present, we not only estimated the association between smoking cessation and smoking-related SCC prognosis but also investigated its potential mechanism. Importantly, we constructed a risk score model combining smoking and HRD, which could effectively screen suitable populations and improve prognostic prediction, especially for smoking-related SCC.

## Materials and Methods

### Patients and Datasets

The data we used were from the public database. Level 3 data of gene expression profiles of LUSC, CSCC, ESCC, and HNSC patients were taken from the GDC Data Portal^1^. Clinical information, including age, gender, stage, tumor status, and survival outcome, were downloaded. The smoking exposure information of each patient was also obtained from The Cancer Genome Atlas (TCGA) database. The HRD scores were obtained from the TCGA Pan-Cancer dataset^2^. All data were extracted from TCGA, an open database, and followed the guidelines. Therefore, there was no requirement for ethics approval.

### Smoking-Related Genes or HRD-Related Genes

Smoking-related genes or HRD-related genes were obtained using the “limma” package in R software. Then, the Kyoto Encyclopedia of Genes and Genomes (KEGG) pathway and Gene Ontology (GO) analysis were performed using the “clusterProfiler” package in R.

### Construction of Risk Score Model in LUSC

Univariable Cox regression analysis was first used to identify survival genes. Then, multivariate Cox regression analysis was again employed to select candidates for building the risk score model. The corresponding coefficients from multivariate Cox analysis were used to calculate risk scores: $s\,c\,o\,r\,e=\sum_{i=1}^{N}\left(E\,x\,p\,i\times C\,o\,e\,f\,i\right)$.

Based on the risk scores, the patients were divided into the high-score group and low-score group. To estimate the prognostic ability of the risk score model, Kaplan-Meier (K-M) survival analysis and survival receiver operating characteristic (survival-ROC) curves were, respectively, applied.

### The Association Between Risk Scores and Prognosis in LUSC

Univariable and multivariable cox analyses were performed to evaluate the association between risk scores and prognosis. Then, a prognostic nomogram was applied as a quantitative tool to accurately predict each patient’s prognosis using risk scores and clinical information. Calibration curves were also plotted to evaluate the accuracy of the nomogram.

### Validation of the Risk Score Model in Other SCC Cohorts

We calculated the risk score in CSCC, ESCC, and HNSC cohorts from the TCGA database based on the same formula. Then, K-M curves and survival-ROC curves were used to estimate the prognostic power and confirm the applicability and reliability of the risk score model in SCC cohorts.

### Validation of the Risk Score Model Using SCC Cohorts From the GEO Database

In addition to the TCGA database, we also used other independent SCC cohorts from the GEO database to verify the risk score model, including the CSCC cohort (GSE44001), ESCC cohort (GSE53625), HNSC cohort (GSE65858), and LUSC cohort (GSE73403). We calculated the risk score of each patient using the same formula. Then, K-M curves and survival-ROC curves were also performed to confirm the prognostic power.

### Statistical Analysis

All data were expressed as mean ± SD (standard deviation). The K-M curves, univariable and multivariable cox analysis were performed using the “survival” package. Survival-ROC curves were applied with “timeROC” the package. The optimal cut-off values of risk scores were evaluated using the “survminer” package. The nomogram and calibration curves were plotted using the “rms” package. The analyses described above were conducted in R software 3.5 and Microsoft Excel 2016.

## Results

### Smoking Cessation and Homologous Recombination

**FIGURE 1:**
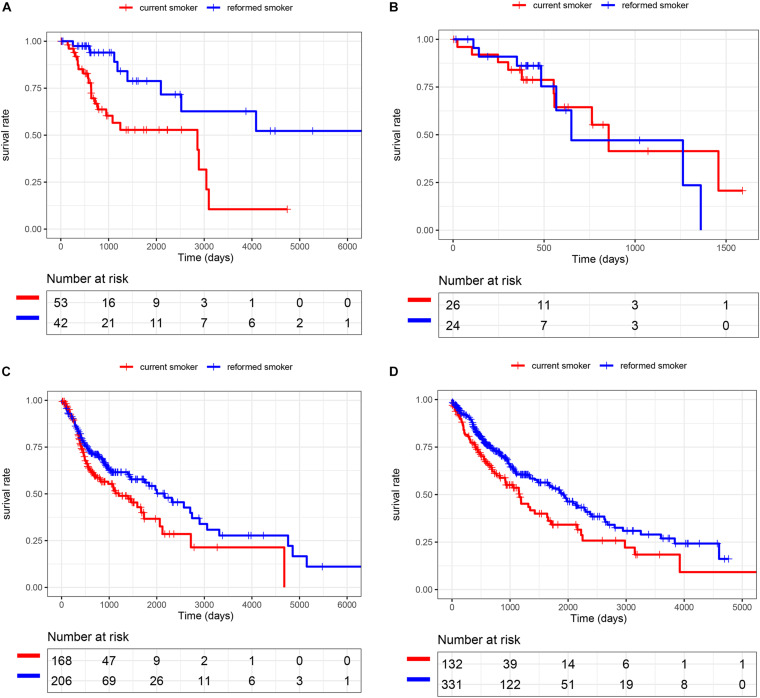
Smoking cessation could improve prognosis in SCC. **(A)** CSCC. **(B)** ESCC. **(C)** HNSC. **(D)** LUSC.

We selected smoking-related SCC patients for further study. A total of 95 CSCC patients (53 current smokers and 42 reformed smokers), 50 ESCC patients (26 current smokers and 24 reformed smokers), 393 HNSC patients (178 current smokers and 215 reformed smokers), and 471 LUSC patients (133 current smokers and 338 reformed smokers) were included in the study. As shown in Figure 1, patients who quit smoking had a longer survival time than those who kept smoking in CSCC (*p* = 0.003), HNSC (*p* = 0.019), and LUSC (*p* < 0.001). Among them, the number of LUSC patients was the largest and the results of survival analysis were the most significant. Therefore, we selected LUSC patients as the training cohort and other SCC cohorts as test cohorts for further study.

**FIGURE 2:**
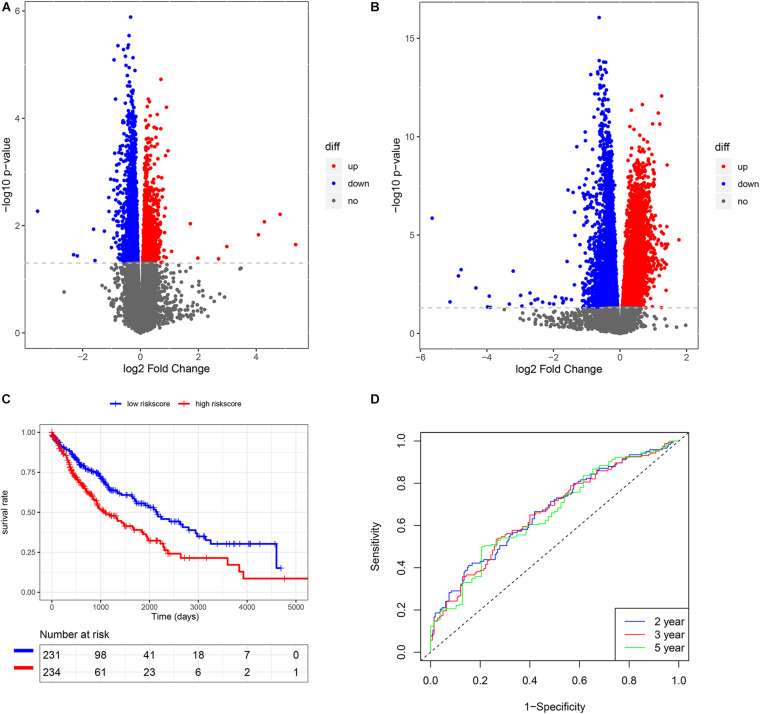
The construction of risk score model in LUSC. **(A)** The volcano plot of smoking-related genes. **(B)** The volcano plot of HRD-related genes. **(C)** K-M analysis of risk score model. **(D)** Survival ROC of risk score model.

**FIGURE 3:**
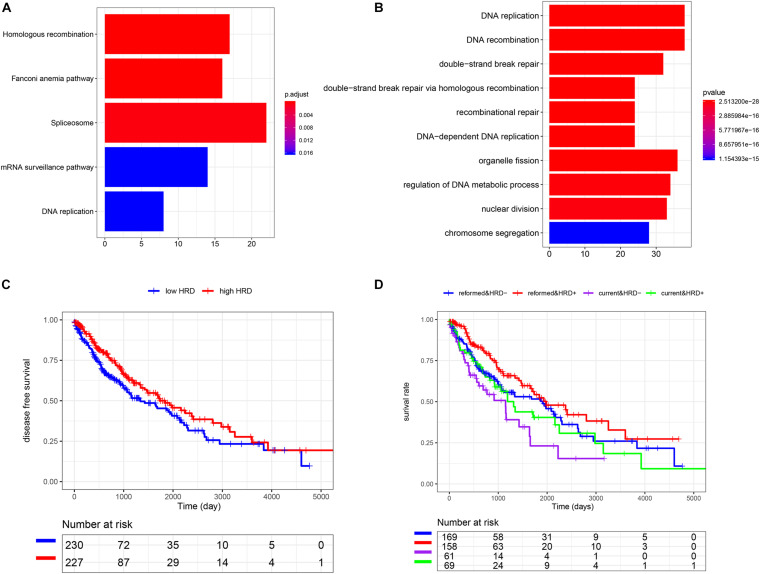
The association among smoking cessation, HRD, and prognosis in LUSC. **(A)** The KEGG analysis. **(B)** The GO analysis. **(C)** K-M analysis of high and low HRD scores. **(D)** The effect of cross-talk between the smoking status and homologous recombination on the prognosis.

To understand the potential mechanism of the benefits of smoking cessation, we obtained smoking-related genes between current smokers and reformed smokers in LUSC patients (Figure 2A). Then, function analysis was performed. KEGG analysis found that homologous recombination (HR) was the most significant pathway in smoking-related LUSC (Figure 3A). GO analysis also found that HR-related GO terms, including double-strand break repair via HR, HR, regulation of double-strand break repair via HR, and negative regulation of double-strand break repair via HR, were significantly enriched (Figure 3B and Supplementary Table 1). Moreover, LUSC patients with higher HRD scores had better survival outcomes than those with lower HRD scores (Figure 3C, *p* = 0.043). We further investigated the effect of cross-talk between the smoking status and HR on the prognosis. LUSC patients were stratified in the combination of the smoking status and HRD scores, including current smoking and high HRD scores, current smoking and low HRD scores, reformed smoking and high HRD scores, and reformed smoking and low HRD scores. The survival analysis revealed that quitting smoking with high HRD scores could significantly improve LUSC patients’ prognosis (*P* < 0.01, Figure 3D).

### Construction of Risk Score Model Using the LUSC Cohort

We also subsequently identified the HRD-related genes between high- and low-HRD score patients (Figure 2B). There were 1218 same genes after taking an intersection for smoking-genes and HRD-genes (Supplementary Figure 1). Afterward, based on the univariate and multivariate Cox regression analysis (Supplementary Tables 2, 3), five eligible genes, including MAFK, LMBRD1, MESDC1, KLHL15, and E2F4, were selected to build a risk score model. Then, we divided the patients into high-risk and low-risk score groups and found that patients with high-risk scores had a worse outcome than those with low-risk scores (*p* < 0.01, Figure 2C). The survival ROC also indicated that the risk score model had good predicting power at 2, 3, and 5 years (AUC = 0.67, 0.67, and 0.66, Figure 2D).

### The Association Between Risk Scores and Prognosis of LUSC

**FIGURE 4:**
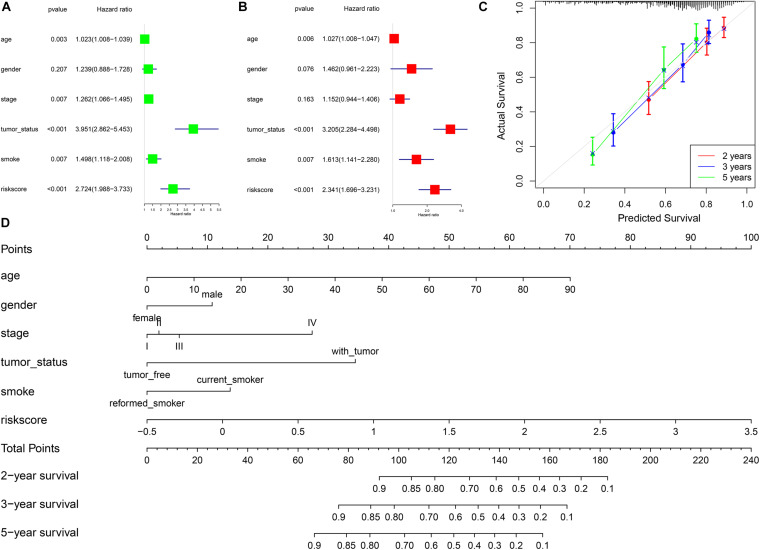
The association between risk scores and prognosis of LUSC. **(A)** Univariate Cox regression analysis. **(B)** Multivariate Cox regression analysis. **(C)** Calibration curve of nomogram. **(D)** Construction of nomogram.

In univariate Cox regression analysis, one risk score could increase the risk of death by 2.72 times (95% CI = 1.99–3.73, Figure 4A) for LUSC patients. A similar, significant increase in death risk was also observed by multivariate Cox analysis (hazard rate, HR = 2.34, 95%CI = 1.70–3.23, Figure 4B), indicating that the risk score model could serve as an independent prognostic indicator. We also provided a prognostic nomogram to predict each patient’s survival of 2, 3, and 5 years, whose calibration curve also suggested its good prediction (c-index = 0.72, 95%CI = 0.71–0.74) (Figures 4C,D).

### Validation of the Risk Score Model in SCC Cohorts

**FIGURE 5:**
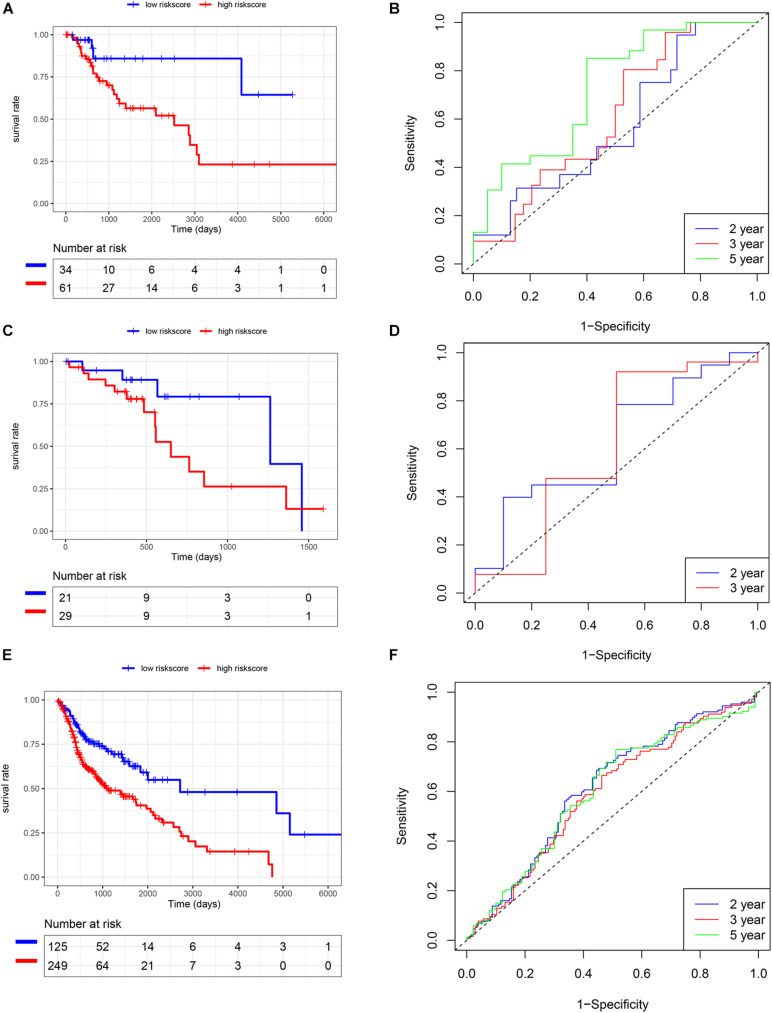
Validation of the risk score model in TCGA SCC cohorts. **(A,B)** CSCC. **(C,D)** ESCC. **(E,F)** HNSC.

We also analyzed the expression level of these five genes in other SCC cohorts and found that most of them were also related to smoking cessation (Supplementary Table 4). Importantly, we estimated the prognostic value of the risk score model in SCC cohorts. K-M and survival-ROC analysis showed that high-score patients had higher hazard rates than low-score patients in CSCC (*p* < 0.01, AUC = 0.60, 0.62, and 0.73 at 2, 3, and 5 years, Figures 5A,B), in HNSC (*p* < 0.01, AUC = 0.62, 0.60, and 0.61 at 2, 3, and 5 years, Figures 5E,F). There was no statistical significance in ESCC patients, but low-score patients tended to live longer than high-score patients (*p* = 0.12, AUC = 0.63 and 0.61 at 2 and 3 years, Figures 5C,D).

### Validation of the Risk Score Model Using SCC Cohorts From the GEO Database

**FIGURE 6:**
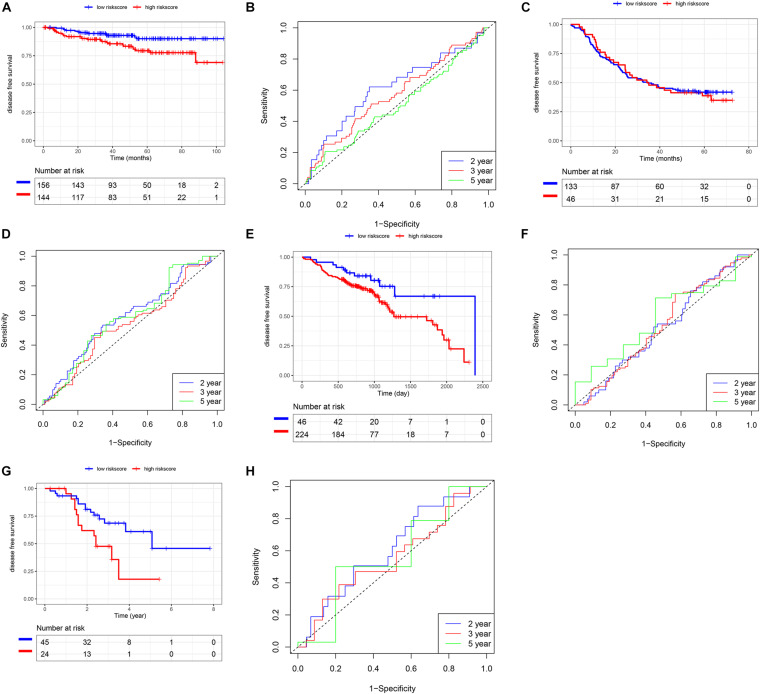
Validation of the risk score model using SCC cohorts from the GEO database. **(A,B)** CSCC. **(C,D)** ESCC. **(E,F)** HNSC. **(G,H)** LUSC.

In addition to the TCGA database, we also use other independent SCC cohorts from the GEO database to verify the risk score model. K-M and survival-ROC analysis showed that high-score patients had higher hazard rates than low-score patients in CSCC (*p* = 0.01, AUC = 0.62, 0.58, and 0.52 at 2, 3, and 5 years, Figures 6A,B), in HNSC (*p* = 0.03, AUC = 0.52, 0.53, and 0.58 at 2, 3, and 5 years, Figures 6E,F), and in LUSC (*p* = 0.02, AUC = 0.61, 0.56, and 0.57 at 2, 3, and 5 years, Figures 6G,H). However, there was no statistical significance in ESCC patients (*p* = 0.86, AUC = 0.59, 0.54, and 0.59 at 2, 3, and 5 years, Figures 6C,D).

## Discussion

Tobacco smoking has been confirmed to be a critical risk factor for SCC development, and smoking cessation can increase overall survival after diagnosis (Dobson Amato et al., 2015; Yavorski and Blanck, 2016). To further understand the benefits of quitting smoking for patients’ prognosis, we selected smoking-related SCC patients from the TCGA database.

By plotting K-M curves, we found that current smokers had a worse prognosis than reformed smokers in most SCC cohorts, which was consistent with other studies (Li et al., 2020; Yang et al., 2020). Then, we found smoking-related genes and performed function analysis in LUSC, pointing out that HR was an important pathway of smoking to aggravate cancer. It is known that DNA double-strand breaks induced by cigarette smoke can be repaired mainly through HR repair (Helleday, 2003; Albino et al., 2004). Many genes involved in HR repair are significantly associated with smoking to influence the risk of lung cancer (Ryk et al., 2006; Nogueira et al., 2010). Hammouz et al. (2020) also constructed a gene co-expression network and found that smoking significantly affected HR to induce lung adenocarcinoma. In addition, many scholars have used HRD scores in cancer studies. Takaya et al. (2020) analyzed the association between HRD score and high-grade serous ovarian carcinoma and suggested that patients could be classified into different prognostic subtypes for personalized treatment. Kraya et al. (2019) investigated the molecular features of BRCA1/2 alterations in breast cancer patients and found that HRD scores and hormone receptor subtypes could predict the immunogenicity of BRCA1/2 breast cancer, and provided the basis for formulating the best immunotherapy strategy. In the present study, we also found that high HRD scores could increase survival time, suggesting that the cross-talk between smoking cessation and high HRD scores might help treatment and improve overall survival.

After the univariate and multivariate Cox regression analysis, the risk score model was established using five smoking- and HRD-related genes in the LUSC cohort, including LMBRD1, MAFK, MESDC1, KLHL15, and E2F4. LMBR1 domain containing 1 (LMBRD1) encodes a lysosomal membrane protein that may be involved in the transport and metabolism of cobalamin. Mutations of LMBRD1 are also associated with vitamin B12 metabolism disorder (Fettelschoss et al., 2017). MAF bZIP transcription factor K (MAFK), an important transcription factor of the MAF family, is associated with epithelial–mesenchymal transition and malignant progression in different cancers (Wang et al., 2015; Okita et al., 2017). Mesoderm development candidate 1 (MESDC1), also known as TLNRD1, is confirmed as an oncogenic function in bladder cancer and hepatocellular carcinoma (Tatarano et al., 2012; Wu et al., 2017). The kelch-like family member 15 (KLHL15) can encode a member of the kelch-like family of proteins and is involved in protein ubiquitination and cytoskeletal organization (Ferretti et al., 2016; Zhou et al., 2019), but few studies focus on cancers. E2F transcription factor 4 (E2F4), a member of the E2F family of transcription factors, plays an important role in inhibiting proliferation-associated genes, and its gene mutation and increased expression are related to different cancers (Gong et al., 2020; Zhuang et al., 2020). In the future, these genes should be deeply investigated roles in SCC. Afterward, we performed K-M and survival-ROC analysis to estimate the predictive value of the risk score model, suggesting that the model was a predictor with good sensitivity and specificity in LUSC patients.

To better predict the prognosis of each patient, we constructed a nomogram integrating risk scores, smoking status, and clinical information. As a practical tool to improve predictive accuracy, these methods have been applied in many studies on different cancers (Sun et al., 2020; Yang et al., 2020). The nomogram with different aspects of markers, including risk scores, smoking status, age, gender, and tumor stage, had high clinical application value, which might be a promising way to change clinical management (Birkhahn et al., 2007).

Lastly, we validated the risk score model in other SCC cohorts from TCGA and GEO databases, indicating that the application of risk scores could be extrapolated to CSCC and HNSC patients.

The present study is of great significance in theory and application. First, we demonstrated that keeping smoking after diagnosis could increase the risk of SCC death, suggesting that smoking cessation should be a part of cancer treatment. Second, the risk score model was constructed using smoking- and HRD-related genes, which could make up for the deficiency of HRD based on BRCA (Turner et al., 2004; Lord and Ashworth, 2016), more effective in screening the population suitable for targeted therapy. In the future, we should further verify the application of the risk score model in different SCC cohorts, especially in Asian or Chinese populations.

## Conclusion

The present study provided comprehensive insights into the association among smoking status, HRD, and prognosis in SCC. Moreover, a risk score model integrating smoking and HRD was constructed to serve as the potential predictive biomarker and add effectiveness in screening the suitable population for targeted therapy.

## Data Availability Statement

The original contributions presented in the study are included in the article/Supplementary Material, further inquiries can be directed to the corresponding author/s.

## Author Contributions

CC, XC, and SL conceived and designed the study, performed the bioinformatic analysis, and wrote the manuscript. HC, MC, LB, and HJ contributed to the revision of the manuscript draft. All authors read and approved the final manuscript.

## Conflict of Interest

The authors declare that the research was conducted in the absence of any commercial or financial relationships that could be construed as a potential conflict of interest.

## Footnotes

1 https://portal.gdc.cancer.gov/

2 https://xenabrowser.net/datapages/
