# Supplementary figures and images for: A Novel Signature for Predicting Prognosis of Smoking-Related Squamous Cell Carcinoma
Source: Front Genet. 2021 Apr 22;12:666371. doi: 10.3389/fgene.2021.666371 (PMC8100348; doi:10.3389/fgene.2021.666371)

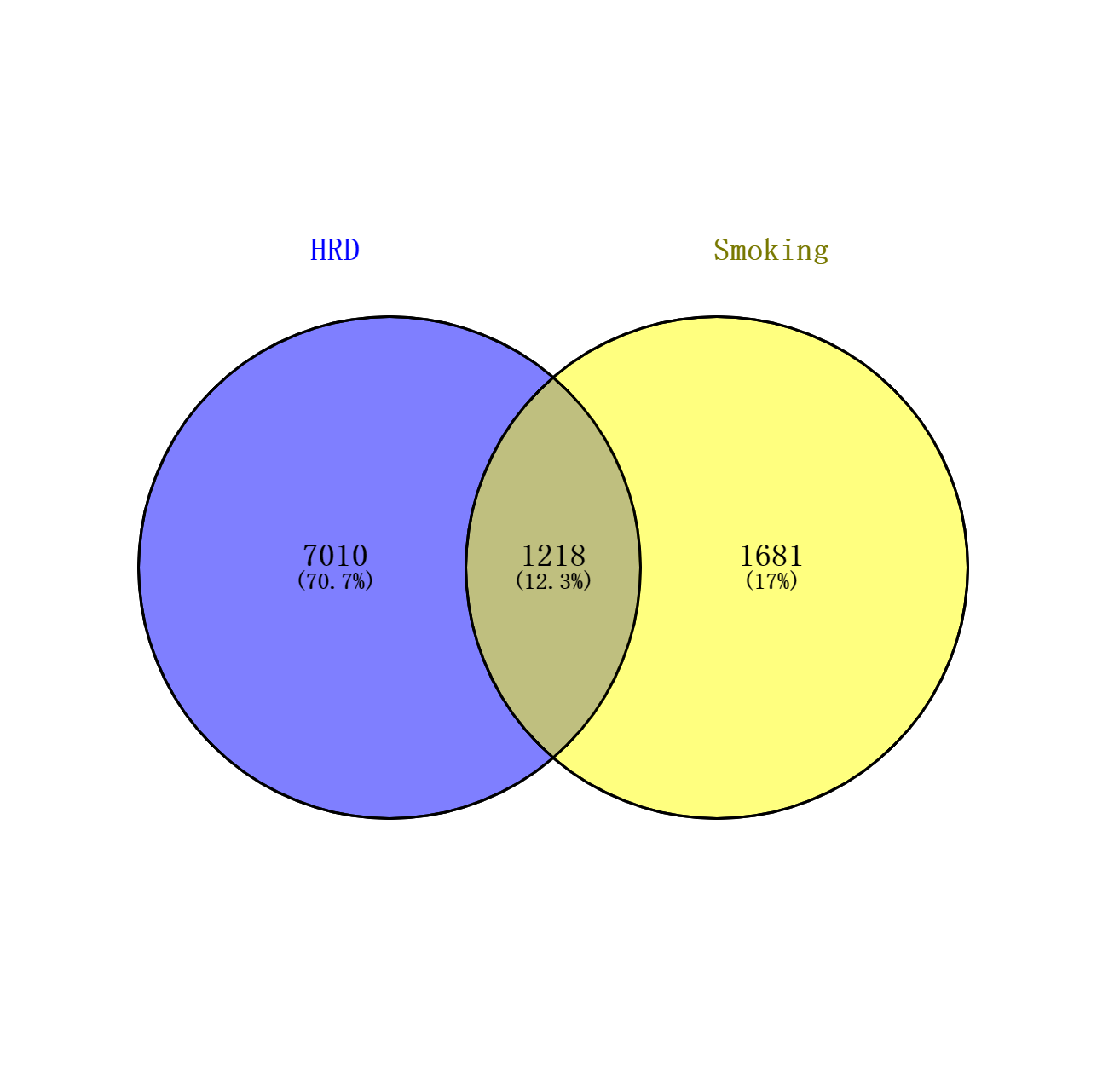

Supplement: Supplementary file 1 [file Image_1.TIF]
