# Supplementary material for: A Novel Signature for Predicting Prognosis of Smoking-Related Squamous Cell Carcinoma
Source: Front Genet. 2021 Apr 22;12:666371. doi: 10.3389/fgene.2021.666371 (PMC8100348; doi:10.3389/fgene.2021.666371)
Supplement: Supplementary file 5 [file Table_4.DOCX]

|  | LUSC | | CSCC | | ESCC | | HNSC | |
| --- | --- | --- | --- | --- | --- | --- | --- | --- |
| Gene | Fold change | P value | Fold change | P value | Fold change | P value | Fold change | P value |
| MAFK | 0.82 | 0.02 | 0.85 | 0.03 | 1.03 | 0.19 | 0.89 | 0.04 |
| LMBRD1 | 1.17 | <0.01 | 1.01 | 0.88 | 1.63 | 0.21 | 0.99 | 0.97 |
| MESDC1 | 0.83 | <0.01 | 0.99 | 0.99 | 1.16 | 0.98 | 0.96 | 0.39 |
| KLHL15 | 0.71 | 0.01 | 1.09 | 0.26 | 1.37 | 0.02 | 0.93 | 0.33 |
| E2F4 | 0.85 | <0.01 | 0.99 | 0.85 | 0.96 | 0.45 | 0.86 | <0.01 |

Supplementary table 4. The expression of five genes (reformed smokers *VS* current smokers) in SCC cohorts
